# The CAPE TOWN modified squat and smile test: correlation with fracture union in long bone fractures of the lower limb

**DOI:** 10.1007/s00590-025-04532-w

**Published:** 2025-11-19

**Authors:** Delroy Arnolds, Sithombo Maqungo, Michael Held, Nando Ferreira, Roopam Dey, Robyn Waters, Maritz Laubscher, Simon Matthew Graham

**Affiliations:** 1 University of Cape Town, Rondebosch, South Africa; 2 Stellenbosch University, Stellenbosch, South Africa; 3 University of Oxford, Oxford, UK

**Keywords:** Fracture union, Intramedullary nailing, Lower limb, Patient-reported outcomes, Outcome

## Abstract

**Purpose:**

Assessing fracture union remains a significant challenge in low-resource settings, such as those across Sub-Saharan Africa. The original squat and smile test was developed as a potential surrogate measure for lower limb fracture union, aiming to reduce reliance on follow-up radiographs in environments with limited access to imaging. We evaluated the correlation between the (blinded) Cape Town modified squat and smile test (CTMSST) and fracture union following intramedullary nailing of lower limb long bone fractures.

**Methods:**

We performed a retrospective review of prospectively collected data from the HIV in Orthopaedic Skeletal Trauma study.

**Results:**

A total of 180 patients with recorded CTMSST data were included in the analysis. There was no significant correlation between the CTMSST total score, or its individual domains (squat, support, and smile), and radiological evidence of fracture union. However, health-related quality-of-life measures (EQ-5D and disability rating index) showed a significant positive correlation with the total CTMSST score, as well as with the squat and smile domains (*p* < 0.05). No significant correlation was found between the support domain and these measures.

**Conclusion:**

The CTMSST and its individual domains did not correlate with radiological fracture union following intramedullary nailing of lower limb fractures. However, the test showed significant positive associations with patient-reported outcome measures, suggesting potential utility in assessing functional recovery. Further prospective research is needed to validate the CTMSST and to explore its role in both clinical assessment and follow-up care in resource-limited settings.

## Introduction

Lower limb long bone fractures are debilitating injuries and are more common in low-to-middle-income countries (LMIC) [1]. In addition to the high burden of these injuries, many LMIC have limited access to resources such as radiological imaging [2]. Assessing fracture union is a critical component of orthopaedic care and is typically performed through clinical examination and confirmed radiologically with X-rays. However, this process poses significant challenges in resource-constrained settings, where patients may be unable to attend regular follow-up appointments and radiographic imaging is often unavailable or prohibitively expensive [3].

An ideal fracture union assessment tool should be quick and easy to use, as well as accurate and cost-effective. It must also have good inter-rater and intra-rater reliability. The ability to use this tool remotely would be of further benefit, especially in resource-constrained environments [4].

A simple functional outcome test to assess mobility and stability of lower limb joints involves assessing a patient’s ability to squat [5]. A squat involves lowering the hips from a standing position while bending the knee and ankle joints and returning to a standing position [6]. This action is one of the most basic functional movements that is required to perform activities of daily living and religious practices. The squat and smile test (SST) was developed as a clinical measure of weight bearing and range of motion (ROM) in LMICs and has been shown to be a possible surrogate for confirming fracture union in lower limb fractures, without the need for radiological imaging [7].

The SST involves assessing a patient performing a deep squat, with as little support as possible, and observing their facial expression. The rationale is that if a patient can hold themselves in the squat position and smile, it shows that they have returned to full function. In addition, deep knee bend shows restored ROM. The squat position mechanically stresses the fracture site, testing that bone fragments have healed. It is suggested that the patient’s smile indicates that they are pain-free [8]. However, a key limitation of the test is that the facial expression component has been shown to correlate poorly with actual fracture union, likely due to its subjective nature [7].

In this study, we used a modified SST hereafter referred to as the CTMSST, in which the "smile" component was adapted to increase objectivity. The primary aim was to evaluate the correlation between the CTMSST and radiographic fracture union following intramedullary nailing of lower limb long bone fractures. Secondary aims included comparison of individual CTMSST component scores with fracture union, as well as with the Radiographic Union Score for Tibial fractures (RUST), the EuroQol-5 Dimension (EQ-5D) health-related quality-of-life score and the disability rating index (DRI).

## Materials and methods

### Study design

We performed a retrospective review of data from the HIV in Orthopaedic Skeletal Trauma (HOST) study database, a multi-centre prospective observational study (NCT03131947) involving patients aged > 18 years with fresh (within two weeks of injury), closed and open tibia and femur fractures who underwent intramedullary (IM) nailing for fracture fixation [9]. Data collection for the HOST study took place between September 2017 and December 2018.

### Study sample

We included data from patients in the HOST study who had their CTMSST recorded and videoed during follow-up appointments following IM nailing for femur and/or tibia shaft fractures. This study was conducted in a challenging environment, and on some occasions, video could not be obtained during clinic review. The absence of video footage was due to logistical factors, rather than patient characteristics, and we do not believe this introduced systematic bias into the findings. Patients without available video footage of the CTMSST were excluded. Additionally, patients who had undergone contralateral or ipsilateral lower limb IM nailing were excluded to eliminate potential confounding factors affecting functional assessment. Informed consent was obtained from all participants for both study participation and video recording.

### Data collection

Data collected from the HOST study database included patient demographic information, CTMSST overall test scores, sub-scores, fracture union as assessed by the RUST, EQ-5D and DRI scores. Additionally, we retrieved video footage of patients at their routine follow-up assessments as part of the HOST study. Clinical assessment, X-rays, EQ-5D, DRI, CTMSST and RUST scores were all recorded at 6 weeks, 3 months, 6 months and 12 months post-operatively. If a participant was confirmed united prior to 12-month follow-up, the scores at union confirmation were used as the final score.

### CTMSST test

**Fig. 1:**
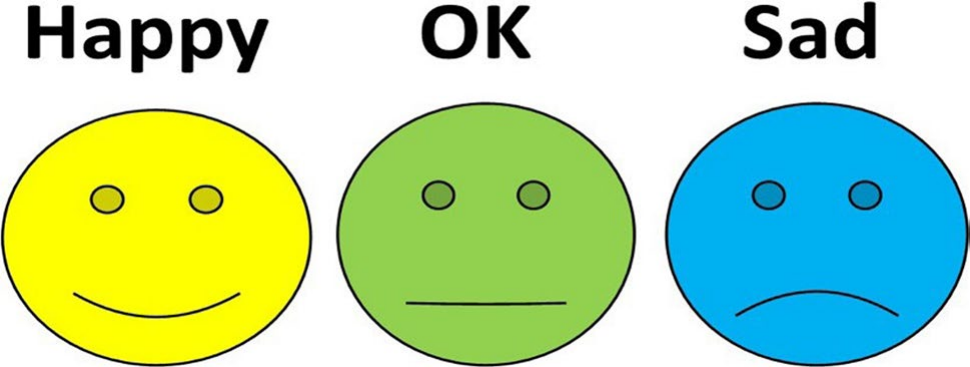
Smiley face (yellow), neutral face (green) and unhappy face (blue) as emoticons patients could select to rate their pain during squatting (colour figure online)

All patients include had their CTMSST videoed by the study team. We assessed the “smile” component of the test by asking patients to point to a picture of a facial expression that best depicts their pain level after performing a squat and by observing dynamic video footage, instead of assessing static photographs, as performed in previous studies (Fig. 1).

The CTMSST test video footage for each patient was viewed by two investigators. One was an orthopaedic trainee, and one was a non-specialist. Investigators graded the squat in the following three main domains (Table 1): overall squat (0 = unable to squat at all, 1 = less than 45 degrees flexion at hip, 2 hips above level of the knees, 3 = hips at level of the knees or below); the need for support (0 = unable to squat unassisted, 1 = requires support with two hands, 2 = requires support with one hand, 3 = no support needed); and smile (1 = sad/frown, 2 = no smile, 3 = smile). The “squat” component of the score was determined to be either “Able to squat” (scores of 2 and 3) or “Unable to Squat” (scores of 0 and 1).

**Table 1:** (Blinded) squat and smile scoring

SST Domain	Score	Explanation
Overall squat	0	Unable to squat at all
	1	Less than 45° of flexion at the hip
	2	Hips above the level of the knees
	3	Hips at the level of the knees or below
The need for support	0	Unable to squat unassisted
	1	Requires support with two hands
	2	Requires support with one hand
	3	No support needed
Smile	1	Sad/frown
	2	No smile
	3	Smile

### Fracture union assessments

Bone healing was assessed using the validated radiological union scoring system for tibia (RUST). Delayed bone union was defined as impaired bone healing at 6 months on RUST score^.^ Non-union was defined as either impaired bone healing at 9 months on RUST score or the need for further surgery to achieve union (RUST score, 9) before 9 months (decision made by two orthopaedic surgeons) [10–12].

Two reviewers (both orthopaedic surgeon - SMG and ML) blinded to CTMSST score, independently assessed radiological fracture union on radiographs. In case of discrepancies in RUST scoring between the reviewers, a third reviewer (orthopaedic surgeon - SMQ) independently undertook a review of the radiographs to determine the outcome.

### Statistical analysis

Descriptive statistics were used to summarise participants’ demographic and clinical characteristics. To evaluate the inter-rater reliability of the CTMSST, the absolute agreement intra-class correlation coefficient (ICC) and 95% confidence interval (CI) were calculated for the three SST domains (squat, support and smile). The ICC measures score reliability by comparing the variability of different scores assigned to the same participant, with the total variation across all scores and all participants. ICC values < 0.50 were categorised as “poor agreement”; 0.50 ≤ ICC < 0.75 as “moderate agreement”; 0.75 ≤ ICC < 0.90 as “good agreement” and ICC ≥ 0.90 as “excellent agreement” [13]. Fisher’s exact test was used to determine the association between fracture union (yes/no), open and closed fractures, and mechanism of injury (MOI).

The Kendall rank correlation coefficient was used to determine the association between components of the CTMSST and age, EQ-5D, DRI and RUST scores. The difference in CTMSST total and sub-domain scores between the union and non-union subgroups was evaluated using the Mann–Whitney U test. All analyses were performed by an independent statistician using IBM SPSS Statistics (version 27) and the level of significance was set at *p* < 0.05.

## Results

**Fig. 2:**
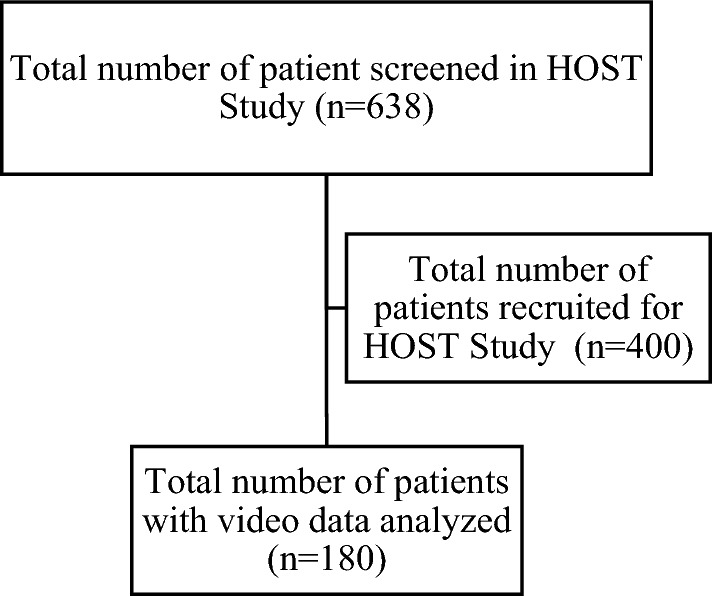
Study flow diagram shows the number of individuals who underwent IM nailing for fracture fixation

Between September 2017 and December 2018, 638 patients underwent IM nailing of the femur and the tibia at the two study sites and were screened for eligibility. A total of 442 IM nails in 400 patients were included in the HOST study. Of these patients, 220 were excluded because a video of CTMSST was not recorded. Final data analysis was performed on 180 participants (Fig. 2).

The demographic and clinical characteristics of the 180 patients are summarised in Table 2.

**Table 2:** Demographic and clinical characteristics of the 180 study participants presenting with lower limb fractures

Variable	Value
*Sex, n (%)*	
Male	144 (80)
Female	36 (20)
Mean age, years (SD)	34.5 (10.6)
*MOI, n (%)*	
PVA	74 (41)
MVA	43 (24)
GSW	31 (18)
High energy fall	11 (6)
Low energy fall	9 (5)
Assault	5 (3)
Crush (heavy machinery)	3 (2)
Blunt force trauma	3 (2)
Other	1 (0)
*Fracture type, n (%)*	
Closed	111 (62)
Open	69 (38)
*Location of fracture and treatment nailing type, n (%)*	
*Femur*	81 (45)
*Right*	*51 (63)*
AFN (antegrade)	39 (76)
RFN (retrograde)	12 (24)
*Left*	*30 (37)*
AFN (antegrade)	25 (83)
RFN (retrograde)	5 (17)
*Tibia*	99 (55)
*Right*	*50 (51)*
SPN (suprapatellar)	44 (88)
IPN (infrapatellar)	6 (12)
*Left*	*49 (49)*
SPN (suprapatellar)	47 (96)
IPN (infrapatellar)	2 (4)
*Union, n (%)*	
Yes	143 (79)
No	37 (21)
Mean EQ-5D index, (SD)	89.69 (12)
Mean DRI score, (SD)	203.5 (159)

DRI, disability rating index; MOI, mechanism of injury; GSW, gunshot wound; MVA, motor vehicle accident; PVA, pedestrian vehicle accident; RUST, radiological union score; SD, standard deviation; AFN, antegrade femoral nail; IPN, infrapatellar nail; RFN, retrograde femur nail; SPN, suprapatellar nail

Inter-rater reliability was excellent for the two components of the CTMSST scored by reviewers (Squat and Support) (Table 3).

**Table 3:** Inter-rater reliability of CTMSST. Mean scores are represented by the intra-class correlation coefficient (ICC) and 95% confidence interval (CI)

CTMSST components	Mean scores		ICC (95% CI)
	Observer 1	Observer 2	
Squat	2.2 (0.9)	2.2 (0.9)	0.971 (0.96–0.98)
Support	2.7 (0.7)	2.6 (0.8)	1.00
Total	7.2 (1.8)	7.2 (1.9)	0.99 (0.97–0.99)

The median CTMSST scores for fractures that were united and non-united fractures were 8 and 7, respectively. The difference was not statistically significant (*p* = 0.113)). There were no significant differences in CTMSST sub-scores between participants with or without fracture union (summarised in Table 4).

**Table 4:** Comparing CTMSST scores between patients with and without fracture union

CTMSST Domains	Union n = 143	Non-union n = 37	*p*-value	Mann–Whitney U test (z)	Cohen effect size (r)
Squat	2 (2–3)	2 (1–3)	0.235	−1.19	0.08
Smile	3 (2–3)	3 (2–3)	0.107	−1.61	0.12
Support	3 (3–3)	3 (3–3)	0.771	−0.29	0.02
Total	8 (6–9)	7 (6–8.5)	0.113	−1.59	0.12

Sensitivity and specificity were 81% and 35%, respectively, while the positive and negative predicative values were 83% and 33%, respectively (Table 5).

**Table 5:** Comparing ability to squat with union/union

	Able to squat (score 2–3)	Unable to squat (score 0–1)	
Union	116 (81%)	27 (19%)	143
Non-union	24 (65%)	13 (35%)	37
Total	140	40	180

Sensitivity 116/143 × 100, Specificity 13/24 × 100, PPV 116/140 × 100, NPPV 13/40 × 100

EQ-5D had a statistically significant correlation with the total CTMSST score (*p* < 0.05), the squat domain (*p* < 0.01) and the smile domain (*p* < 0.05). EQ-5D had no correlation with the support domain.

DRI had a statistically significant inverse correlation with the total CTMSST score (*p* < 0.001), squat domain (*p* < 0.001) and smile domain (*p* < 0.001). DRI had no correlation with the support domain.

Age had an inverse correlation with all three components of the CTMSST. All these associations were statistically significant (*p* < 0.05).

RUST score had no correlation to the total CTMSST score, squat domain and support domain.

The association between RUST and the smile component was significant (*p* < 0.05) (Table 6).

**Table 6:** Kendall’s tau correlates with the CTMSST

Variable	(Blinded) squat and smile test			
	Squat	Support	Smile	*Total*
EQ-5D score	0.16**	0.02	0.13*	0.10*
DRI score	−0.30***	−0.02	−0.27***	−0.26***
Age	−0.25***	−0.16*	−0.14*	−0.25***
RUST score	0.13	0.10	0.21**	0.12

*p < 0.05. **p < 0.01. ***p < *0.001*

*p < 0.05 -> statistically significant at the 5% level

*p < 0.01 -> statistically significant at the 1% level

*p < 0.01 -> statistically significant at the 0.1% level

## Discussion

This study aimed to determine the correlation between the (blinded) Cape Town modified squat and smile test(CTMSST) and fracture union of lower limb long bone fractures, post-intramedullary nailing. We modified the smile domain of the CTMSST, as it was subjectively assessed and prone to physician bias. We attempted to make the smile component more objective, and we hypothesised that the modified score may correlate better with fracture healing. In our study, we found no correlation between the CTMSST and fracture union. The test demonstrated a positive association with patient-reported outcome measures, indicating its potential value in assessing functional recovery. Further prospective studies are required to validate the CTMSST and to determine its role in clinical assessment and follow-up care, particularly in resource-limited settings.

Currently, the combination of clinical history assessment with comprehensive examination, and supplementation with radiological imaging, is used to assess fracture healing. Previous studies have assessed the relationship between fracture union and the squat and smile, most of them from the SIGN (Surgical Implant Generation Network) Fracture Care database [14–16]. In 2017, Eliezer et al*.,* assessed the SST and its correlation with fracture union and found no correlation between the two [17]. In 2019, Wu et al*.,* defined the domains of the squat and smile test and found that the squat and support domain correlated with the need for reoperation. The most common reasons for reoperation in their study were infection (15/272) and non-union (3/272). Sciuto et al*.,* found the squat and smile test correlated with union, but not with the smile domain [14]. However, their cohort included paediatric patients and used a different scoring system for fracture union (REBORNE). They also used a different scoring system for the squat and smile (GAS) and not the one originally proposed.

In our study, quality-of-health measures in the form of EQ-5D and DRI had a significant correlation with total CTMSST score, squat domain and smile domain. The support domain had no correlation.

The smile domain of the CTMSST correlated with quality-of-life measures, a finding which highlights the previous subjectivity of this component and the need for patient-driven assessment tools and outcome measures. This was expected as both elements take the patient’s views and scores of their overall recovery status into account.

This retrospective review has several limitations. We used data from an existing dataset that determined the study population. Our sample size was limited, and a power calculation was not performed for analysis. The findings should therefore be interpreted with caution. The availability of CTMSST video recordings was not prespecified in the HOST protocol, and the findings should therefore be interpreted with caution. Despite no standardisation of how videos were taken, our study showed good reliability of the CTMSST. Future studies would benefit from a larger, prospectively collected dataset.

## Conclusion

We found no correlation between fracture union of lower limb fractures post-intramedullary nailing and the CTMSST, as well as its sub-scores. However, we observed a strong correlation between quality-of-life measures and the CTMSST, particularly in the squat and smile domains. Notably, the association between the RUST score and the smile component was significant, suggesting that if a patient can smile during the test, it may reflect a level of recovery comparable to radiographic healing. These findings highlight the potential role of the CTMSST in assessing functional recovery and patient-reported outcomes, rather than as a surrogate for fracture union. Further research is warranted to refine the CTMSST and to validate its utility in larger, prospective cohorts.

## Data Availability

Data cannot be shared openly but are available on request from authors.
